# Developing Medical Technologies for Low-Resource Settings: Lessons From a Wireless Wearable Vital Signs Monitor–neoGuard

**DOI:** 10.3389/fdgth.2021.730951

**Published:** 2021-10-15

**Authors:** Assumpta Nantume, Sona Shah, Teresa Cauvel, Matthew Tomback, Ryan Kilpatrick, Bushra Afzal, Noah Kiwanuka

**Affiliations:** ^1^Neopenda, PBC, Chicago, IL, United States; ^2^Floating Children's Hospital at Tufts Medical Center, Boston, MA, United States; ^3^Department of Biostatistics and Epidemiology, Makerere University School of Public Health, Kampala, Uganda

**Keywords:** vital signs, wearable sensors, wireless health monitor, newborn health, digital health, medical technology

## Abstract

The neoGuard™ technology is a wireless wearable vital signs monitor attached to a patient's forehead to continuously measure oxygen saturation, pulse rate, respiratory rate and temperature. Developed with feedback from more than 400 health workers, primarily in East Africa, the product has been designed to meet the unique constraints of low-resource settings. This perspective piece by the innovators of neoGuard™ and some of their key partners examines the complicated journey of taking a medical technology from concept through clinical validation and finally to market. By shedding light on some of the most critical steps and common challenges encountered along the pathway to commercialization, the authors hope that their experiences will provide some valuable insights to other aspiring innovators in this space.

## Introduction

In the aftermath of the COVID-19 pandemic, digital health products have gained increasingly wide appeal for the delivery of health services to patients in high-income and low-income settings alike. As researchers, clinicians and policy makers rallied to optimize patient care and alleviate the burden on health facilities and hospital staff at the height of the pandemic, global efforts to leverage digital health solutions became more invigorated than ever before (1–3).

Yet despite the recent uptick in digital health interest, innovators still struggle to navigate the existing ecosystem and face significant barriers to transitioning their ideas from concept to market (4), particularly in low-and-middle-income countries (LMICs) where the health technology space is maturing at a slower pace (5). At the same time, there is a tremendous opportunity for innovators worldwide to seize this unprecedented moment and capitalize on these recent digital health gains. In this perspective piece, the team behind the wireless vital signs monitor, neoGuard, reflects on its journey creating and commercializing a health technology product for patients in low-resource settings. By examining our own experience, we aim to offer some useful insights on potential pitfalls in medical device innovation and how to successfully navigate the current digital health ecosystem.

## neoGuard Origin Story

The idea for Neopenda's neoGuard device started with a mission of addressing an unmet need for vital signs monitoring solutions for newborns in low resource settings. Through a series of formal and informal needs assessments, we quickly established that conventional vital sign monitoring equipment were unable to meet the constraints of low-resource settings due to (i) the high cost of initial purchase and maintenance, (ii) infrastructure challenges such as space limitations and power outages, and (iii) their reliance on single-use accessories which are not always readily available.

Designing a product to adequately meet our user's needs while overcoming these salient challenges became the focus of our innovation efforts. Like many medical technologies and digital health solutions, Neopenda started in the academic world as a design project by a team of graduate biomedical engineering students. Driven by the mission and neoGuard's potential for positive impact on healthcare in underserved communities, we transitioned from academia to a startup venture. After consulting with more than 400 health workers, primarily in Uganda, we were able to design an affordable, reusable, wearable, multi-parameter vital sign measurement device over the course of 4 years.

**Figure 1:**
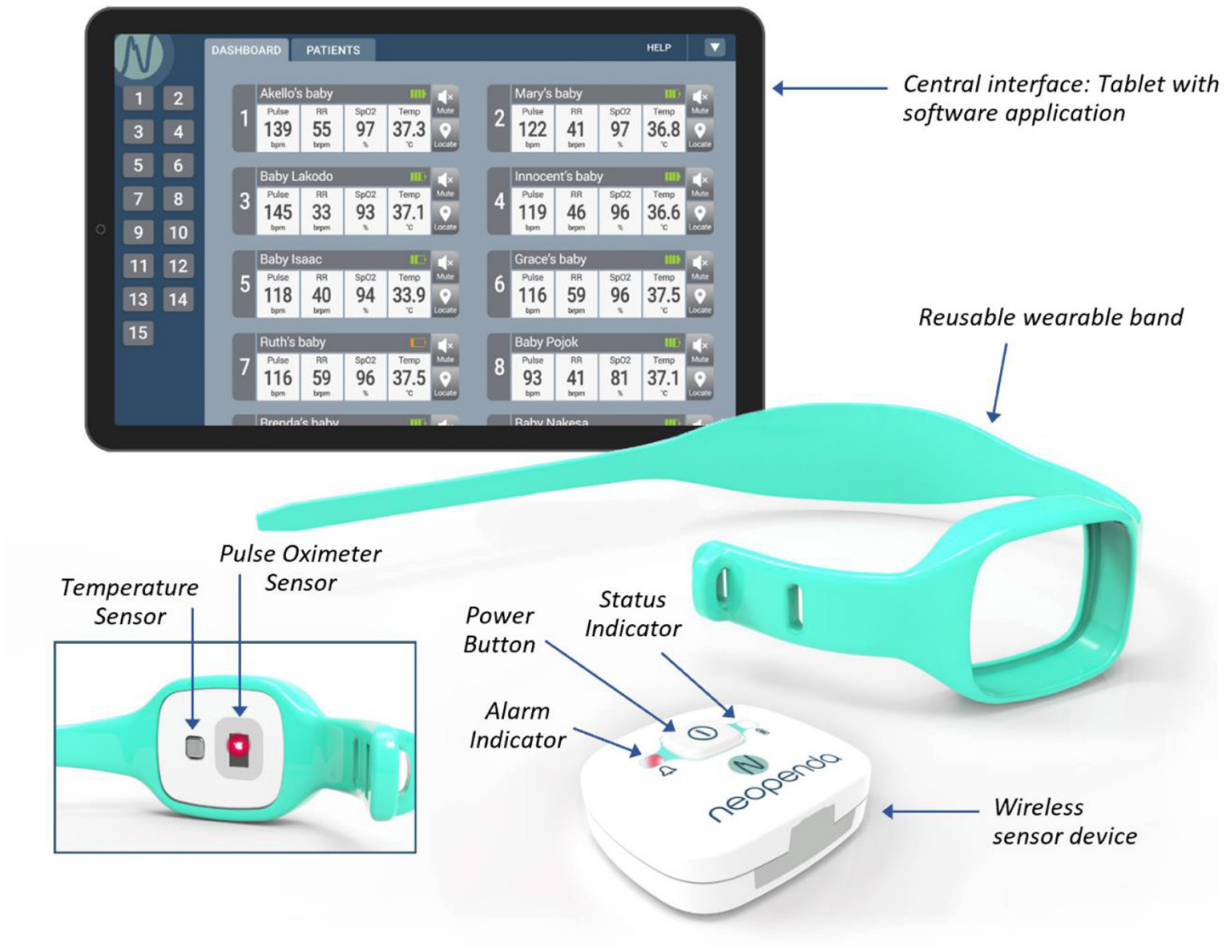
The neoGuard V1.0 vital signs monitoring system.

The neoGuard device continuously measures temperature (temp), blood oxygen saturation (SpO_2_), pulse rate (PR) and respiratory rate (RR), and wirelessly transmits readings to a central dashboard (NeoMonitor app) that is hosted on a tablet (Figure 1). The system provides real-time visual and audio alerts whenever a patient's vital sign measurements fall outside preset upper or lower limits. The NeoMonitor app can display data from up to 15 devices at a time, making it an ideal tool to simultaneously monitor multiple patients in health facilities with very low nurse-to-patient ratios.

## Early Testing

Between November 2018 and November 2019, we conducted two IRB-approved pilot studies that aimed to (i) evaluate the preliminary safety of neoGuard by describing and quantifying any adverse events, (ii) evaluate the ability of the device sensors to detect high fidelity PPG (photoplethysmogram) waveform signals from the newborns' capillaries, and (iii) assess the preliminary concordance of neoGuard measurements with reference measurements from a standard-of-care/conventional vital signs monitor. The pilot studies involved 22 stable newborns (aged <28 days) at Tufts Children's Hospital (formerly Floating Hospital for Children) at Tufts Medical Center in Boston, Massachusetts and 27 healthy newborns and infants (aged <16 weeks) at Jinja Regional Referral Hospital (Jinja RRH) in Jinja, Uganda. Regulatory approvals were obtained from the Tufts Health Sciences Campus Institutional Review Board for the Tufts study, and from the Makerere University School of Public Health (MakSPH) Higher Degrees Research and Ethics Committee and the Uganda National Council of Science of Technology for the Jinja study.

Results from both studies showed that the neoGuard device demonstrated comparable safety to standard-of-care equipment (with no adverse events recorded from either system) and sufficient signal quality to register changes in the PPG waveforms. However, accuracy of vital sign measurements was variable from patient to patient, showed high sensitivity to motion artifacts and device placement, and was further impacted by the large size of the prototype hardware device and inadequate design of the headband. Through an iterative design process, we recalibrated sensor settings and refined the vital signs algorithms to reduce noise (unruly signals) from these sources until we attained robust performance for the measurement of temperature, pulse rate, blood oxygen saturation, and respiratory rate. The hardware of the neoGuard device was also optimized, including reduction of the size by over 60%.

**Figure 2:**
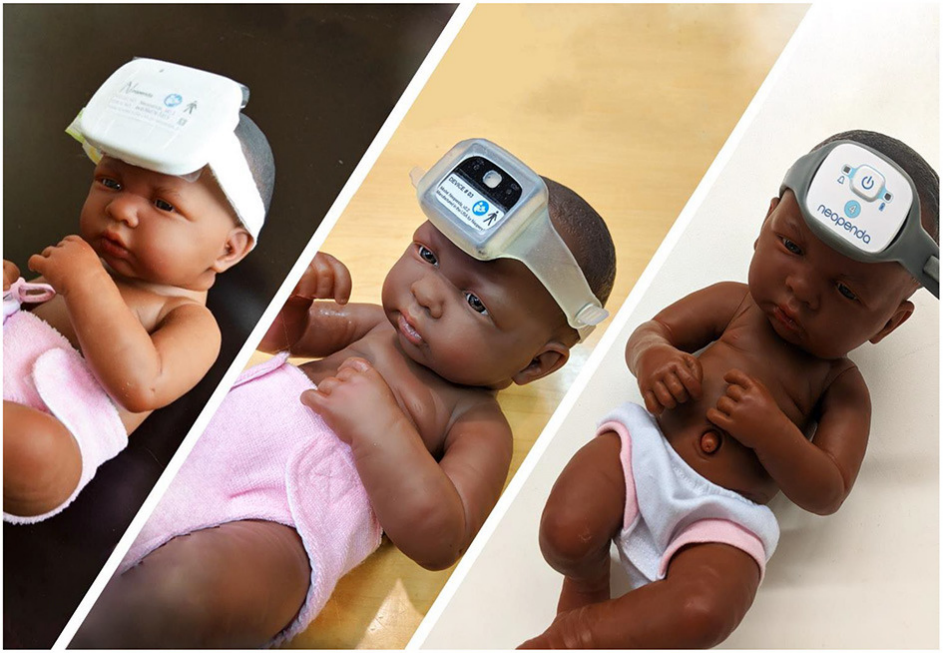
Prototypes of neoGuard hardware versions v1.0 (left), v2.2 (center), and v3.0 (right).

**Figure 3:**
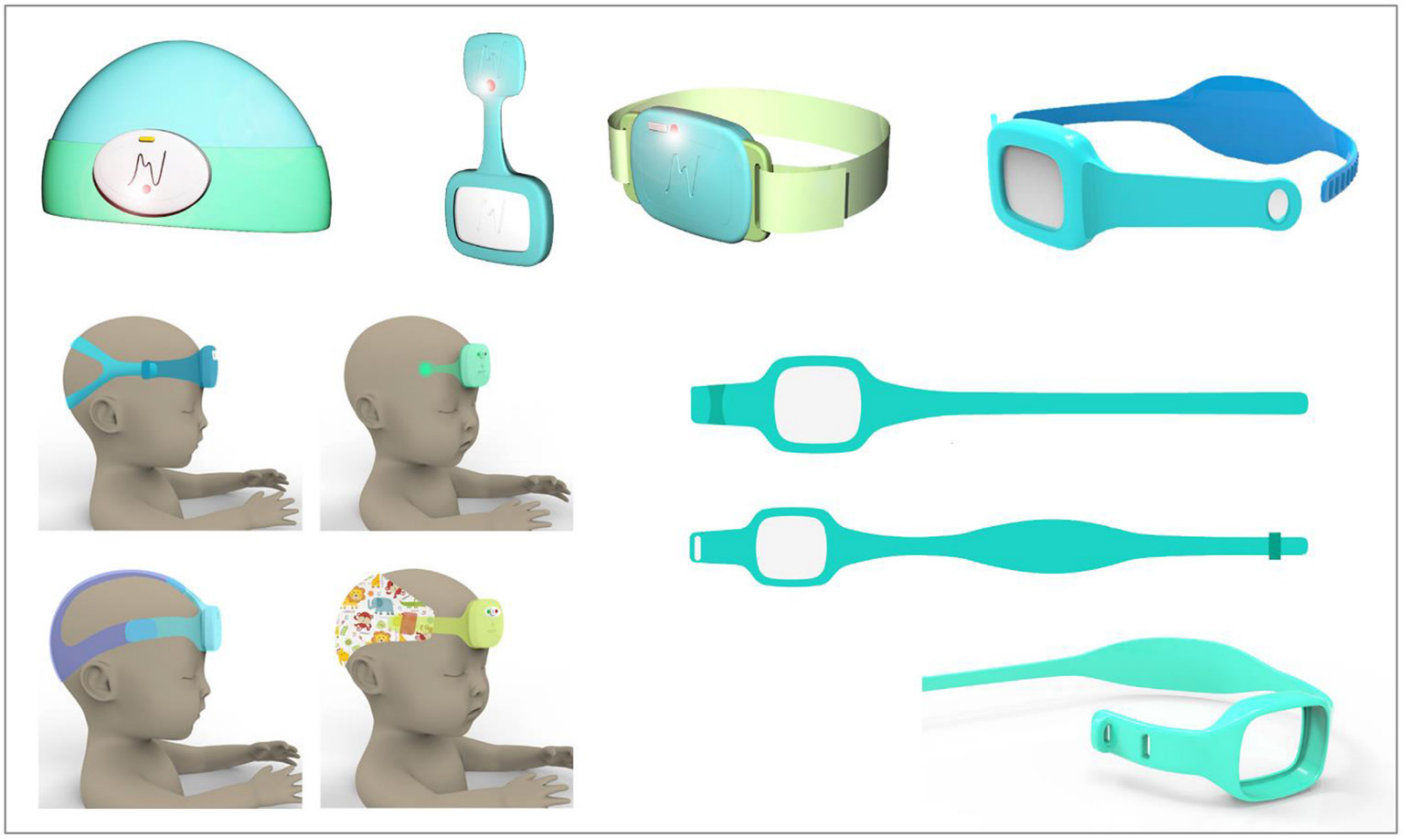
Sampling of neoGuard headband design concepts.

The design process involved three main hardware iterations, culminating in the first production release of the device, neoGuard wearable device (Figure 2). Feedback from clinicians involved in the Tufts and Jinja studies was also invaluable for the improvement of the neoGuard headband design. The band evolved from a disposable fabric band, to a two-piece silicone band, to an adjustable single-piece reusable silicone band (Figure 3). Later, an extender strap was created to enable the headband to fit adult patients as well. Being responsive to stakeholder feedback throughout the development process is crucial to creating a product that will be readily adopted by end users. Without this iterative, collaborative early phase, neoGuard would have performed less successfully in the human factors usability engineering studies and post-market clinical follow-up studies later on.

In addition to performance testing, early research on neoGuard also explored human factors and usability engineering (HFUE). HFUE, an essential component of the medical device design and development process, involves bringing users and stakeholders into the design process to make sure that the solution being created will meet their needs and be usable for them. Data on HFUE was collected through training workshops, simulation exercises and user surveys, and interviews. This HFUE data drives many design decisions and product requirements. In total, ~70 health staff participated in the formative and summative assessments for HFUE. An additional 330 health staff constituted the voice-of-customer (VOC) research.

## Clinical Validation Studies

To comply with ISO/IEC standards for pulse oximetry (6) and temperature monitoring (7) across the appropriate measurement ranges, final accuracy validation was conducted at Clinimark Laboratories in Louisville, CO, USA and the Hypoxia Lab at University of California San Francisco (UCSF). All testing was conducted with production-equivalent units, that is, the “final” product or equivalent. Testing on human subjects was conducted under IRB approvals from Clinimark Laboratories and UCSF.

We attained robustperformance for measurement of temperature (range 30–40°C, accuracy ±0.3°C, over a variety of ambient conditions), pulse rate [range 45–205 beats per min (bpm), accuracy ±3 bpm], blood oxygen saturation (range 70–100%, accuracy ±4%), and respiratory rate [range 5–30 breaths per min (brpm), ±5 brpm]; meeting the accuracy requirements of the applicable ISO/IEC standards (Table 1).

**Table 1:** Summary of clinical validation study results.

**Vital sign**	**Accuracy**	**Resolution**	**Claimed range**	**Sample size (# subjects)**	**Sample size (data points)**	**Validation method**	**Study dates, sites**
PR	±3 bpm	1 bpm	Baby mode: 75–205 bpm Adult mode: 45–145 bpm	N/A	50	Functional bench testing using an electronic pulse simulator	September 2020, Clinimark
RR	±5 brpm	1 brpm	5–30 brpm	20	655	Clinical respiratory rate study in comparison to end tidal carbon dioxide monitor	September 2020, Clinimark
SpO_2_	±4%	1%	70–100%	13	275	Clinical hypoxia desaturation study in comparison to arterial co-oximetry	August 2020, UCSF Hypoxia Lab
Temp	±0.3°C Equilibration time ≤ 10 min	0.1°C	30.0–40.0°C	N/A	15	Functional bench testing using a NIST traceable fluid bath	October 2020, Clinimark

## Regulatory Approval

As a medical device used in the clinical environment, neoGuard is subject to rigorous regulation in all markets to ensure it is safe and effective. To create a medical device meeting the standards for commercial, clinical use, a medical device company must implement a design control process, and quality management system in compliance with internationally recognized standards such as ISO 13485 (8). Early in the design and development process the applicable regulatory standards for the product should be identified and incorporated into the product requirements. If the design team does not have regulatory or quality expertise, external consultants are necessary. Neopenda began contracting regulatory and quality expertise in year 2.

In accordance with the CE mark classification guidelines, we categorized the neoGuard product as a class IIb medical device and pursued the CE marking regulatory pathway (route 1) to demonstrate compliance with the European Union Medical Device Regulation (EU MDR) (9). Under this process, we underwent a full quality assurance audit by a notified body and obtained ISO 13485:2016 certification for our quality management system. Next, we submitted a technical file for the neoGuard product to the same notified body. After review of the technical file and audits of both Neopenda and our critical sub-contractors, the EU certificate was granted. Then the CE mark and notified body number are affixed to the neoGuard product and a Declaration of Conformity is ratified.

CE mark certification is recognized by most medical device regulators in our target countries (10) and will allow us to bring the neoGuard product to market efficiently. In addition to CE mark, many countries also require appointment of a local agent/representative to complete product registration with the appropriate regulatory body and obtain final import and marketing clearance.

## Commercialization Efforts

To reach most hospitals in emerging markets, medical equipment is often procured through a wholesale distributor. In Kenya and Uganda, where Neopenda has launched neoGuard, we have worked closely with a local authorized representative and distributor to register the neoGuard product and obtain import clearance from the Kenya Pharmacy and Poisons Board and the National Drug Authority, respectively.

Successful adoption of a new technology extends beyond the design of a product, and into appropriate implementation. This includes an emphasis on user training, installation, preventative and reactive maintenance, and adequate sales and marketing efforts. Our immediate strategy consists of leveraging the expertise of local distributors to implement neoGuard. This model provides a scalable approach to penetrating new markets without having to build a large sales/marketing team in every country we enter into. Similarly, on the production side, we have outsourced manufacturing of the neoGuard devices to a contract manufacturer with the infrastructure, supplier network and quality management system necessary to mass produce, package, and deliver thousands of devices to our in-country distributors efficiently and cost-effectively.

## Post-Market Performance Evaluation

Under the EU MDR requirements, it is recommended that medical device innovators complete at least one post-market clinical follow-up (PMCF) study after launching their product. In addition to the PMCF study, innovators are also encouraged to initiate broader scope evaluations encompassing a larger number of users/patients in order to supplement their pre-market approval findings on the safety and effectiveness of the approved device. Clinical data from post-market studies are not only valuable for post-market surveillance. These studies maybe referenced in updating claims on device efficacy, expanding the product's indications for use, as well as assessing market acceptance and uptake (11).

Neopenda has plans to implement at least six post-market research studies on the neoGuard product, including short-term implementation feasibility studies in Uganda, Kenya, Tanzania, Nigeria, and Mali, and a medium-to-long term clinical impact and cost-effectiveness study in Kenya. At least two of the planned studies will be led by independent non-profit partners in our target countries.

In parallel with our formal research, we will also conduct routine monitoring and evaluation where we aim to (i) capture monthly or quarterly performance metrics through the neoGuard backend data repository and (ii) gather additional user feedback through routine surveys with early adopters.

## Potential Pitfalls and Lessons Learned

### Pre-mature Product Testing

While there is an understandable temptation for innovators to begin testing their products as soon as they have a functional prototype, clinical research is an expensive endeavor, and even small scope pilot studies have considerable time and cost implications. It is important to plan out the phases of testing that will be needed, and to first generate sufficient evidence that your product will perform as intended before you put it to the test in a formal clinical setting. Where possible, this can be achieved through bench top testing in a lab setting; for instance, with the neoGuard product, we were able to employ a pulse rate simulator and water bath to test for pulse rate and temperature, respectively, before further testing on human subjects. It is also important to note that significant product modifications may arise from the early testing phase while you conduct thorough bench testing and gather user feedback. In some cases, it might require more than one pilot study before you are fully confident in initiating your design freeze. The “final” clinical validation study(s) must be conducted after design freeze, with the final or production-equivalent product. While the introduction of new devices requires testing within the target market, one bottleneck that innovators are likely to run into in LMICs is the limited access to gold-standard equipment or measurement techniques. For instance, our research team had intended to use end tidal carbon dioxide (EtCO_2_) as a comparison method for the respiratory rate measurement by neoGuard; however, EtCO_2_ monitors were not readily available in Uganda, so we opted to perform this component of testing with a US-based researched partner. As far as possible, innovators in low-resource settings should leverage partnerships with institutions that may have better access to the resources they need to perfect and test their early prototypes. This will significantly help reduce the risk of testing a product in-field before it is fully functional or ready to be tested by users. That said, the importance of early product testing in target markets should not be overlooked however complicated the process may be. Environmental considerations such as temperature, humidity, or the layout of health facilities can have a significant impact in the performance of your technology, and gathering this data early on will enable innovators to respond proactively.

### Insufficient Preparation for Quality Management and Regulatory Affairs

A medical device should be designed within the existing regulatory framework and quality requirements. Innovators should know the product specifications that are deemed acceptable in clinical practice, as well as any electrical safety, electromagnetic compatibility (EMC), or radio frequency emissions standards pertaining to their product. Your target parameters and corresponding validation tests needed should be clearly defined in your technical file. Conduct extensive research on the regulatory pathway you are pursuing and engage experienced personnel and/or consultants to help you navigate any policy hurdles. It is important to be familiar with the IRB process and documentation that may be necessary. However, the IRB process for research involving medical devices is often not well-defined and is still evolving in many low-resource settings. Having IRB-approval from a US-based institute or other country where medical device studies occur more frequently may help strengthen your efforts for research clearance with a local IRB. In many cases, local IRBs may not have the necessary medical device expertise to adequately assess the risks and benefits or your research, so providing evidence of review by another IRB can help quail some of their concerns. In the same regard, selection of an IRB committee with relevant experience reviewing medical device research is critical. Some of the essential documents you should prepare to include in your research application are: a detailed protocol, operator's manual, investigator's brochure, informed consent forms, and preliminary safety and efficacy data. For devices that are above non-significant risk, which do not pose significant risk to human subjects, pre-approval and registration with the country's medical product regulatory agency may be necessary. It is important to be familiar with secure data ownership and security requirements to protect subject privacy and health information. After initial IRB approval, studies will be subject to regular reviews and any significant protocol or device changes will require IRB approval. Having the right expertise on your team will help limit the amount of time it takes to reach your next milestone. In preparing your budget, anticipate that the quality and regulatory stage will almost definitely cost more and take longer than you project.

### Applying Inappropriate or Impractical Research Methods

Think carefully about your choice of research methods and consider your justification for why your methodology is appropriate. The nature of a medical device, the context in which it is applied, and cost implications can often limit the study design to more simplistic single-arm trials. Regardless of the underlying reasons, it is important to reflect on the limitations of any study design. Seek out opportunities to collaborate with research partners from academia or non-profits who are working to address similar challenges. Collaborators may provide expert knowledge and external insights on the feasibility of your product.

Ensure that your data collection methods and data analysis approach are equally robust. Where possible, validate or pre-test your research tools for clarity, face validity, and content validity. Provide relevant training and guidance to study personnel involved in data collection. Research objectives should place equal emphasis on both the quantitative performance metrics and the qualitative user experience. A comprehensive analytical framework should also take into consideration the role of social forces (e.g., trust in medical authorities, myths around technologies) and how the requirements or expectations of a medical product may vary in different clinical situations and cultures. For instance, many users of the neoGuard technology shared concerns that the patients caretakers or family may feel anxious seeing a device affixed to a patients forehead, as this was an unusual attachment area for a vital sign monitor. However, when it came to actual implementation of the technology, we observed that this anxiety was not as prevalent as we had imagined. Patients' caretakers and family demonstrated a high level of trust in the medical staff attending to their patient, and the medical staff reported that they had no trouble explaining the role of the technology and responding to any questions or concerns that patients or caretakers harbored. Through user surveys and caretaker interviews, we concluded that the unusual placement of neoGuard on the forehead of the patient would be acceptable.

Finally, innovators must consider if the evidence they are generating will satisfy the requirements of their target audience. For instance, while local stakeholders might appreciate results from a familiar clinical setting within the target market, regulators will more often be inclined to accept performance findings from studies conducted in a more controlled lab setting or by an accredited third party with the ability to test your device against acceptable standards. As discussed previously, access to the acceptable standards maybe more limited in low-resource environments.

### Lack of Emphasis on Human Factors and Usability Engineering

Focusing heavily on the performance aspects of the technology without considering how human factors can lead to user errors and how this might impact the safety and effectiveness of the product can yield unfavorable results. At Neopenda, we believe strongly in the theory of human-centered design, and we leverage VOC and HFUE data to drive design decisions. This is more important than ever for products intended for low-resource environments; sustainability of the design is essential, and the product must be long-lasting and easy to implement and use. When conducting VOC research, it is necessary to capture perspectives from all categories of users. For instance, for a phone-based application, the ability to correctly use the technology will be influence by factors such as age, smart-phone ownership, and/or experience and computer literacy. These factors will vary not only from person to person but from location to location. If a technology *s* intended to be implemented broadly in rural and urban facilities alike, then the VOC and HFUE data should be collected from all relevant settings.

Elements of VOC and HFUE are often conducted early on in the product development lifecycle. As a result, they are poorly documented and loosely structured. It is crucial to create some sort of guiding framework for your VOC and HFUE, however, flexible and imperfect it might be. For instance, if user testing in a real setting is not immediately practical, then consider how you might best simulate a testing environment or script hypothetical scenarios for users to respond to. To best understand what challenges or barriers users may encounter in using your technology, you want to recreate the same user experience as closely as possible to measure any recurrent errors. In a sense, your goal is to approach the user testing like a controlled experiment, leaving room for variability only at the user level. On the subject of documentation, innovators should record these early efforts in real-time and incorporate them in a Design History File (DHF). A DHF should systematically encapsulate every critical design change and all relevant feedback or data that helped inform that change. Tracing this pathway to the final product is important not only from a regulatory perspective, but also to remind the innovator of why certain design choices were made in case future product changes threaten to impact them. For Neopenda, our approach to VOC and HFUE was not clearly defined when we started; in fact, it took months of retrospective analysis and piecing together various sources of data gathered over the course of 3 years to map out how human factors had been accounted for in relevant design features.

### Inadequate Stakeholder Engagement

Engagement with key stakeholders is required throughout the medical device life cycle. Failing to adequately consult the full breadth of potential stakeholders may have negative effects such as costing the innovator additional time and finances if any important early processes are skipped. Buy-in from various stakeholders—users, ministries of health, international non-governmental organizations, implementation partners, and hospital administrators, amongst many others—is essential to success. Getting buy-in can be challenging particularly at the early stages of development, and building relationships takes time. Even once a stakeholder or partner is engaged, management of the partner and regular communication is key to sustaining their interest and support. Setting clear expectations of goals, responsibilities, timelines, and financial responsibilities is essential for continued engagement. While synergizing efforts between stakeholders can yield more efficient and effective results, managing stakeholders can become a time-consuming and challenging task if expectations are not set in advance and maintained throughout the relationship by both parties. Cultural differences will also influence how well stakeholders respond to your medical product and how your motivations are perceived. Foreign innovators coming into low-resource settings with only minimal experience in the subject they are addressing are likely to be met with far greater skepticism than innovators who have a solid foundation working in their target communities. This is why innovation hubs cropping from familiar entities like non-profits or academia appear to have a more positive experience implementing new products or strategies in LMICs than traditional startups operating on their own. To add to that, however well-meaning an innovator's intentions may be, it is difficult for stakeholders to ignore the commercial incentive behind an enterprise. In this regard, non-profit and academic actors tend to wield more power and influence in the innovation space in LMICs because they are generally perceived to be more altruistic than traditional startups. This is another reason why individual innovators should consider collaborating or having their products independently evaluated by reputable non-profit or academic partners. Working with stakeholders who have no commercial conflicts and are able to speak independently regarding the performance of a product will give stronger credibility to an innovator's claims.

### Unreliable Pre-market and Post-market Revenue Streams

Taking a medical device from idea to commercialization is an arduous and iterative process that requires significant upfront capital. Innovators may elect to receive funding through non-dilutive capital (e.g., grants or competitions) and/or dilutive capital (e.g., through accelerators or investors), and the type of capital received is likely to evolve as the company matures. Planning and budgeting for the full life cycle of a medical device is a challenging but important task; many innovators fail to receive enough funding to get through the life cycle. Unlike many software or service provider startups, medical device development requires significant upfront capital. The capital is required to fund R&D efforts such as prototyping, field testing, clinical validation, scale-up to manufacturing (e.g., tooling costs to produce the device at scale), general operating expenses such as salary, rent, and legal fees, and regulatory and quality activities. Early-stage medical device innovators are often perceived as a high-risk investment due to the length of time it takes to get a medical device to market, and the upfront capital required before generating revenue. Defining a roadmap and associated budget can demonstrate an innovator's expertise in medical device development. Execution of the roadmap with acceptable pivots but within the planned budget will de-risk the company as innovators progress with their roadmap.

Beyond the initial financing for product development, innovators should have a clear understanding of the business environment(s) they are entering into and how long it may take before they are able to generate a steady stream of revenue. Evidence that there is a need for a product does not guarantee that the market will readily take up the product once it is available. In pursuing new leads, innovators should be conscious not to overestimate their market size as well as their customer's willingness and ability to pay. To establish some initial brand recognition and help kick-start market penetration for your technology, it is worthwhile to approach potential consumers or customer segments early on in the product development process to understand not only how they respond to your product, but how they evaluate and make purchasing decisions. In the buildup to the launch of the neoGuard product, Neopenda completed more than 80 hospital visits, and engaged with numerous implementing partners, health officials and health distributors across East Africa as part of our customer acquisition plan. It is important to remember that the end-users of a medical product and the buyers are usually two different entities. An effective marketing strategy should present a complete value proposition to appeal to both parties.

## Conclusion

While many medical device innovators aspire to transition their concepts through regulatory approval and mass production to widespread adoption, very few are able to make it across that finish line. The journey is fraught with setbacks and challenges that unfortunately leave many promising ideas sidelined or abandoned.

Through our own experience developing the neoGuard product, we have learnt that the process takes an exceptional amount of effort, agility, resilience, patience, funding, and overall–teamwork. The myriad of problems that innovators face can be successfully managed or even avoided through interdisciplinary collaboration. To increase the likelihood that a solution will be actualized, innovators should surround themselves with engineering, clinical, manufacturing, regulatory, and marketing expertise at an early stage of their product lifecycle.

## Data Availability Statement

The raw data supporting the conclusions of this article will be made available by the authors, without undue reservation.

## Ethics Statement

The studies involving human participants were reviewed and approved by Makerere University School of Public Health Higher Degrees Research Ethics Committee and the Tufts Medical Center Institutional Review Board. Written informed consent to participate in this study was provided by the participants' legal guardian/next of kin.

## Author Contributions

AN and SS discussed the lessons learned. TC, MT, and RK wrote content for the background on product development, early testing, and clinical validations studies. BA and NK contributed to the introduction and clinical components of the paper. All authors contributed to the final manuscript.

## Funding

This project received funding from the Efficiency for Access Research and Development Fund (RD0002) and Grand Challenges Canada (1911-30987).

## Conflict of Interest

TC and SS are the innovators of the neoGuard product and co-founders of Neopenda, PBC. AN and MT are employed by Neopenda, PBC. This project received funding from the Efficiency for Access Research and Development Fund. The funder had no involvement in the product development or research efforts discussed here. The remaining authors declare that the research was conducted in the absence of any commercial or financial relationships that could be construed as a potential conflict of interest.

## Publisher's Note

All claims expressed in this article are solely those of the authors and do not necessarily represent those of their affiliated organizations, or those of the publisher, the editors and the reviewers. Any product that may be evaluated in this article, or claim that may be made by its manufacturer, is not guaranteed or endorsed by the publisher.

## Abbreviations

Bpm: Beats per minute
Brpm: Breaths per minute
COVID-19: Coronavirus disease 2019
DHF: Design History File
EMC: Electromagnetic Compatibility
EU MDR: European Union Medical Device Regulation
HFUE: Human Factors Usability Engineering
IEC: International Electrotechnical Commission
IRB: Institutional Review Board
ISO: International Organization for Standardization
LMICs: Low-and-middle income countries
MakSPH: Makerere University School of Public Health
NBCU: Newborn Care Unit
PMCF: Post-Market Clinical Follow-up
PR: Pulse Rate
RR: Respiratory Rate
RRH: Regional Referral Hospital
SpO_2_: Blood oxygen saturation
UCSF: University of California San Francisco
VOC: Voice-of-Customer.

## Glossary

**CE mark**

An administrative marking that indicates conformity with health, safety, and environmental protection standards for products sold within the European Economic Area.

**Class IIb medical device**

A category for medical devices with medium-to-high risk, such as incubators, long-term corrective contact lenses, surgical lasers, vital sign equipment, and defibrillators.

**Design Controls**

Interrelated practices and procedures that are incorporated into the product design and development process, i.e., a system of checks and balances. Design Controls increase the likelihood that the design transferred to production will translate into a device that is appropriate for its intended use. For medical devices, the rigor of the Design Controls is prescribed by ISO 13485.

**Design Freeze**

The point in the design and development process at which the product is considered “done” and the baseline design is completed. Under ISO 13485-guided medical device development, changes to the design after the Design Freeze point are subject to engineering change control requirements such as traceability, impact analysis, risk assessment, verification, and validation.

**Design History File (DHF)**

A compilation of documentation that describes the design history of a finished medical device.

**Declaration of Conformity**

It is a formal declaration by a manufacturer, or the manufacturer's representative, that the product to which it applies meets all relevant requirements of all product safety directives applicable to that product.

**Human Factors Usability Engineering (HFUE)**

The application of knowledge about human behavior, abilities, limitations, and other characteristics to the design of medical devices (including software), systems and tasks to achieve adequate usability of the end product.

**Technical File**

A set of documents that describes a product and can prove that the product was designed according to the requirements of a quality management system.

**Voice-of-Customer (VOC)**

Customer's feedback about their experiences with and expectations of a product.
